# Oxidative Stress and Inflammatory Biomarkers for Populations with Occupational Exposure to Nanomaterials: A Systematic Review and Meta-Analysis

**DOI:** 10.3390/antiox11112182

**Published:** 2022-11-04

**Authors:** Xiaogang Luo, Dongli Xie, Jianchen Hu, Jing Su, Zhebin Xue

**Affiliations:** 1 College of Textile and Clothing Engineering, Soochow University, 199 Ren-Ai Road, Suzhou 215123, China; 2 Shanghai Institute of Spacecraft Equipment, 251 Huaning Road, Shanghai 200240, China

**Keywords:** nanomaterials, occupational exposure, oxidative stress, inflammation, biomarkers, meta-analysis

## Abstract

Exposure to nanomaterials (NMs) is suggested to have the potential to cause harmful health effects. Activations of oxidative stress and inflammation are assumed as main contributors to NM-induced toxicity. Thus, oxidative stress- and inflammation-related indicators may serve as biomarkers for occupational risk assessment. However, the correlation between NM exposure and these biomarkers remains controversial. This study aimed to perform a meta-analysis to systematically investigate the alterations of various biomarkers after NM exposure. Twenty-eight studies were found eligible by searching PubMed, EMBASE and Cochrane Library databases. The pooled results showed NM exposure was significantly associated with increases in the levels of malonaldehyde (MDA) [standardized mean difference (SMD) = 2.18; 95% confidence interval (CI), 1.50–2.87], 4-hydroxy-2-nonhenal (HNE) (SMD = 2.05; 95% CI, 1.13–2.96), aldehydes C6-12 (SMD = 3.45; 95% CI, 2.80–4.10), 8-hydroxyguanine (8-OHG) (SMD = 2.98; 95% CI, 2.22–3.74), 5-hydroxymethyl uracil (5-OHMeU) (SMD = 1.90; 95% CI, 1.23–2.58), o-tyrosine (o-Tyr) (SMD = 1.81; 95% CI, 1.22–2.41), 3-nitrotyrosine (3-NOTyr) (SMD = 2.63; 95% CI, 1.74–3.52), interleukin (IL)-1β (SMD = 1.76; 95% CI, 0.87–2.66), tumor necrosis factor (TNF)-α (SMD = 1.52; 95% CI, 1.03–2.01), myeloperoxidase (MPO) (SMD = 0.25; 95% CI, 0.16–0.34) and fibrinogen (SMD = 0.11; 95% CI, 0.02–0.21), and decreases in the levels of glutathione peroxidase (GPx) (SMD = −0.31; 95% CI, −0.52–−0.11) and IL-6 soluble receptor (IL-6sR) (SMD = −0.18; 95% CI, −0.28–−0.09). Subgroup analysis indicated oxidative stress biomarkers (MDA, HNE, aldehydes C6-12, 8-OHG, 5-OHMeU, o-Tyr, 3-NOTyr and GPx) in exhaled breath condensate (EBC) and blood samples were strongly changed by NM exposure; inflammatory biomarkers (IL-1β, TNF-α, MPO, fibrinogen and IL-6sR) were all significant in EBC, blood, sputum and nasal lavage samples. In conclusion, our findings suggest that these oxidative stress and inflammatory indicators may be promising biomarkers for the biological monitoring of occupationally NM-exposed workers.

## 1. Introduction

The global nanotechnology market is estimated to reach USD 350.8 Billion by 2025 [1]. The wide production and use of nanomaterials (NMs) lead to increased occupational exposures in workers of many factories and research laboratories [2,3]. Considerable evidence from animal studies shows that NM exposures can cause damages in various organs and induce the development of related diseases (e.g., pulmonary fibrosis [4], lung cancer [5], mesothelioma [6,7], liver fibrosis [8], chronic nephritis [9], myocardial infarction [10], and Parkinson’s disease-like [11]). However, there is a general delay of many years between occupational NM exposures and the development of diseases in human. To prevent the adverse outcomes resulting from long-term occupational exposures and a substantial economic burden from the treatment of them, it is strongly recommended to give biological monitoring for the populations exposed to NMs to realize early warning.

Although the mechanisms associated with injuries induced by NMs are complex, preclinical studies suggest activations of oxidative stress and inflammatory responses are important contributors [12,13]. Thus, oxidative stress- and inflammation-related indicators may be potential biomarkers for occupational risk assessment. This hypothesis has been demonstrated by some epidemiological studies. Wu et al. observed the levels of the lipid peroxidation product 8-isoProstaglandin F2α (8-isoPGF2a) in exhaled breath condensate (EBC) and urine samples as well as urinary total 8-isoprostane were significantly higher in the workers handling NMs (particularly carbon nanotubes, CNTs) than those in non-exposed controls [14]. Pelclova et al. detected the levels of oxidative stress markers malondialdehyde (MDA), 4-hydroxy-trans-hexenale (HHE), 4-hydroxy-trans-nonenale (HNE), 8-isoPGF2a, 8-hydroxy-2-deoxyguanosine (8-OHdG), 8-hydroxyguanosine (8-OHG), 5-hydroxymethyl uracil (5-OHMeU), o-tyrosine (o-Tyr), 3-chlorotyrosine (3-ClTyr), 3-nitrotyrosine (3-NOTyr) and aldehydes C6-C11 in the EBC were significantly elevated in NM workers compared with control subjects [15,16]. Zhang et al. reported the levels of inflammatory cytokines interleukin (IL)-1β, IL-6, IL-8, macrophage inflammatory protein-1β (MIP-1β) and tumor necrosis factor α (TNF-α) were significantly increased in the NM-exposed workers compared with the controls [17]. Unfortunately, inconsistent results were also described by some authors. Ursini et al. found the lack of a statistical significance in biomarkers of oxidative stress (levels of MDA, HNE, 8-Isprostane in EBC as well as 8-OHdG in urine) and inflammation (serum IL-6 and IL-8) between NM-exposed and non-exposed office workers [18]. Zhao et al. found there was no significant difference in the level of IL-1β between NM-exposed and control groups [19]. These inconsistent findings were considered to be associated with small sample sizes causing low statistical power in individual studies.

To provide strong evidence, we aimed to conduct a meta-analysis of the discrepant data obtained from all currently available published studies to systematically evaluate the associations between NM exposure and the levels of various oxidative stress- and inflammation-related indicators. Our results may be useful to screen the markers with the monitoring significance for NM-exposed occupational workers.

## 2. Materials and Methods

### 2.1. Search Strategy

This meta-analysis was executed according to the preferred reporting items for systematic reviews and meta-analysis (PRISMA) 2020 checklist [20]. The electronic databases of PubMed, EMBASE and Cochrane Library were searched to identify related studies published before August 2022, without language restriction. The combination of search keywords included (“employees” OR “workers” OR “professionals” OR “workplace” OR “office” OR “occupational exposure”) AND (“nanomaterials” OR “nanoparticle” OR “carbon nanotube” OR “graphene” OR “quantum dot”) AND (“biomarker” OR “inflammation” OR “immune” OR “oxidative”). The reference lists of all relevant studies and previous reviews were also reviewed to avoid the potential for missing eligible publications.

### 2.2. Inclusion and Exclusion Criteria

Studies were included according to the population, exposure, comparators, outcomes and study design (PECOS) criteria: (a) populations: workers involved in manufacturing and/or handling NMs; (b) exposures: exposure to production or manufacturing area of NM factories and laboratories; (c) comparators: employees without occupational exposure to NMs; (d) outcomes: oxidative stress- and inflammation-related biomarkers. Oxidative stress biomarkers included MDA, superoxide dismutase (SOD), glutathione peroxidase (GPx), HNE, HHE, 8-Isprostane, 8-isoPGF2a, 8-OHdG, 8-OHG, 3-ClTyr, 5-OHMeU, o-Tyr, 3-NOTyr and aldehydes C6-C12. Inflammation biomarkers included fraction of exhaled nitric oxide (FENO), IL-1β, IL-4, IL-5, IL-6, IL-6 soluble receptor (IL-6sR), IL-8, IL-10, TNF-α, MIP-1β, monocyte chemoattractant protein-1 (MCP-1), nuclear factor kappa-B (NF-κB), myeloperoxidase (MPO), C-reactive protein (CRP), club cell secretory protein 16 (CC16), surfactant protein A (SP)-A, SP-D, fibrinogen, vascular cell adhesion molecule (VCAM), intercellular adhesion molecule (ICAM), leukotriene (LT)-B4, LT-C4, LT-D4, LT-E4 and neutrophils; and (e) study design: observational (cohort, panel or cross-sectional).

The exclusion criteria were: (a) duplications; (b) non-original studies (e.g., case reports, reviews, conference abstracts, letters and protocols); (c) preclinical studies (in vitro and in vivo); (d) control groups that consisted of individuals who were not occupationally exposed to NMs were unavailable; (e) data of interest could not be extracted from the included articles; and (f) irrelevant topics. Eligible studies were independently selected by two researchers and disagreements were resolved by the third researcher.

### 2.3. Data Extraction

Two reviewers independently completed data extraction. The extracted information included the first author, country, publication year, study design, sample size, study subjects of exposed (including exposure duration in workplaces) and non-exposed groups, the type of exposed NMs and the sample type utilized in the analysis of outcomes and outcome measures. The results were expressed as mean and standard deviation (SD); if 95% confidence interval (CI), interquartile range, and minimum and maximum values were provided, they were converted to SD appropriately. The data in the figures were extracted by using the GetData Graph Digitizer version 2.26 (GetData Pty Ltd., Kogarah, Australia). Any divergences were resolved by discussion with the third author.

### 2.4. Quality Assessment

The quality of included observational studies was assessed by the Newcastle–Ottawa Scale (NOS) [21]. NOS consisted of eight items that were categorized into three domains: selection of study groups, comparability of groups and determination of exposure. If the answer was yes, one star was assigned for each item (except of the comparability group item, which was given two stars). The total NOS scores ranged from 0 to 9 by adding up all the stars, and studies with a NOS score >6 were considered to be of high quality. Quality assessment was performed independently by two reviewers; any disagreements were resolved by a third reviewer.

### 2.5. Statistical Analysis

Extracted data were stored in a Microsoft Excel file and exported to STATA version 15.0 (Stata Corp., College Station, TX, USA) for the statistical analysis. Since all biomarkers were measured as continuous variables, the standardized mean difference (SMD) and 95% CI were used to express the effect size. The significance of the total SMD was examined by the Z-test and two-sided *p* < 0.05 denoted a statistically significant association of biomarkers with NM exposures. The heterogeneity among studies was assessed by the Cochran-Q test and I-squared (I^2^) statistics. *p*-values < 0.1 or I^2^-values > 50% indicated the presence of heterogeneity. A random effects model was used to pool the estimates if significant heterogeneity was observed; otherwise, a fixed effects model was utilized. Subgroup analyses stratified by NM types and sample sources were conducted to explore the effects of the study characteristics (potential sources of the heterogeneity). The likelihood of publication bias (with over-reporting of positive results and under-reporting of negative results in studies) was appraised with the Egger’s linear regression test (briefly, the standard normal deviate was regressed against the estimate’s precision) [22]. If the publication bias was encountered (indicated by *p* < 0.05), the trim-and-fill method [23] was applied to adjust the publication bias and further ascertain the influence of the publication bias on the outcomes of the meta-analysis. The stability of the meta-analysis results was also confirmed by a sensitivity analysis with a leave-one-out method [24] (that is, each study was omitted at a time and then the pooled estimates were reassessed. The reassessed results were compared with the original results to judge whether the removed studies would alter the pooled results).

## 3. Results

### 3.1. Literature Search

**Figure 1:**
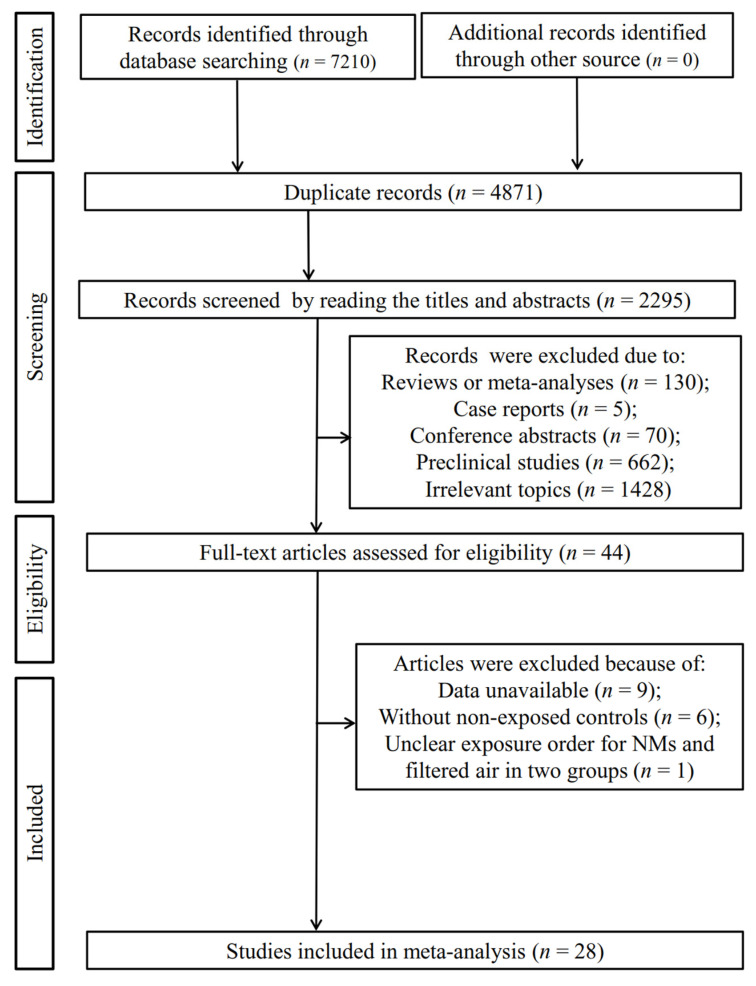
Flowchart of the literature search for the meta-analysis.

As shown in Figure 1, the electronic database search retrieved 7210 records. After the removal of 4871 duplicates, 2339 studies underwent the title and abstract screening, which resulted in 2295 of them excluded because they were reviews or meta-analyses (*n* = 130), case reports (*n* = 5), conference abstracts (*n* = 70), preclinical studies (*n* = 662) and irrelevant topics (*n* = 1428). The remaining 44 studies were entered into a full-text screening to further assess their eligibility. As a result, 16 studies were excluded because of data unavailable (*n* = 9), without non-exposed controls (*n* = 6) and unclear exposure order for NMs and filtered air in two groups (*n* = 1). Eventually, 28 studies with 2636 participants (including 1374 exposed and 1262 non-exposed) were included in the meta-analysis [14,15,16,17,18,19,25,26,27,28,29,30,31,32,33,34,35,36,37,38,39,40,41,42,43,44,45,46].

### 3.2. Study Characteristics and Quality Assessment

The main characteristics of each included article are summarized in Table 1. The publication years of these 28 studies ranged from 2014 to 2022. Ten studies were conducted in China (including five in mainland and five in Taiwan), nine in Czech, two in Netherlands and one in the USA, Italy, Latvia, Russia, Korea, Israel and Australia, respectively. Except of two studies that had a panel design, the other studies used a cross-sectional design. The sample size of all studies was small (*n* < 100 in 19 studies; *n* < 200 in six studies and *n* > 200 in three studies). Most of the participants worked in a workplace producing NMs for more than one year (except some without clear descriptions) and the results may theoretically reflect the long-term exposure effect. Some studies specifically stated the NM type for occupational exposure, such as graphene, silica oxide nanoparticles (SiO_2_NPs), iron oxide nanoparticles (IONPs), titanium dioxide nanoparticles (TiO_2_NPs), indium tin oxide nanoparticles (ITONPs), multi-walled carbon nanotubes (MWCNTs), while others did not provide the detail and were categorized as mixed NMs. All studies attempted to explore non-invasive biomarkers in blood, urine, sputum, nasal lavage or EBC samples. All of the included studies were deemed to be of high quality because the NOS score was 7, 8 or 9 (Table 1).

### 3.3. Meta-Analysis Results

The number of experimental datasets for meta-analysis was larger than the actual number of included articles because multiple NM types, detection time points and sample sources were included for some studies. The detailed data that were extracted for each variable are presented in Table S1.

#### 3.3.1. Association between Occupational NM Exposure and Oxidative Stress Biomarker Levels

A total of 31, 19, 17, 17, 9, 26, 35, 75, 32, 8, 33, 33, 26, 6 and 60 experimental datasets reported the association of NM exposure with the levels of oxidative stress-related indicators MDA, SOD, GPx, HNE, HHE, 8-Isprostane, 8-isoPGF2a, 8-OHdG, 8-OHG, 3-ClTyr, 5-OHMeU, o-Tyr, 3-NOTyr, individual and total aldehydes C6-C12, respectively (Table 2). The pooled analysis showed that compared with the non-exposed group, occupational exposure to NMs was significantly correlated with increases in the levels of MDA (SMD = 2.18; 95% CI, 1.50–2.87; *p* < 0.001), HNE (SMD = 2.05; 95% CI, 1.13–2.96; *p* < 0.001), HHE (SMD = 4.27; 95% CI, 2.13–6.40; *p* < 0.001), 8-Isprostane (SMD = 1.13; 95% CI, 0.76–1.50; *p* < 0.001), 8-isoPGF2a (SMD = 1.22; 95% CI, 0.83–1.60; *p* < 0.001), 8-OHdG (SMD = 1.00; 95% CI, 0.79–1.21; *p* < 0.001), 8-OHG (SMD = 2.98; 95% CI, 2.22–3.74; *p* < 0.001), 3-ClTyr (SMD = 4.36; 95% CI, 2.56–6.16; *p* < 0.001), 5-OHMeU (SMD = 1.90; 95% CI, 1.23–2.58; *p* < 0.001), o-Tyr (SMD = 1.81; 95% CI, 1.22–2.41; *p* < 0.001), 3-NOTyr (SMD = 2.63; 95% CI, 1.74–3.52; *p* < 0.001), aldehyde C6 (SMD = 5.53; 95% CI, 3.29–7.77; *p* < 0.001), aldehyde C7 (SMD = 3.53; 95% CI, 1.83–5.23; *p* < 0.001), aldehyde C8 (SMD = 3.46; 95% CI, 1.48–5.45; *p* = 0.001), aldehyde C9 (SMD = 4.88; 95% CI, 2.69–7.06; *p* < 0.001), aldehyde C10 (SMD = 4.80; 95% CI, 2.93–6.66; *p* < 0.001), aldehyde C11 (SMD = 2.30; 95% CI, 1.16–3.44; *p* < 0.001), aldehyde C12 (SMD = 1.75; 95% CI, 0.77–2.73; *p* < 0.001), total aldehydes C6–C12 (SMD = 3.45; 95% CI, 2.80–4.10; *p* < 0.001) and reductions in the levels of SOD (SMD = −0.24; 95% CI, −0.44–−0.03; *p* = 0.024) and GPx (SMD = −0.31; 95% CI, −0.52−0.11; *p* = 0.003) (Table 2).

**Figure 2:**
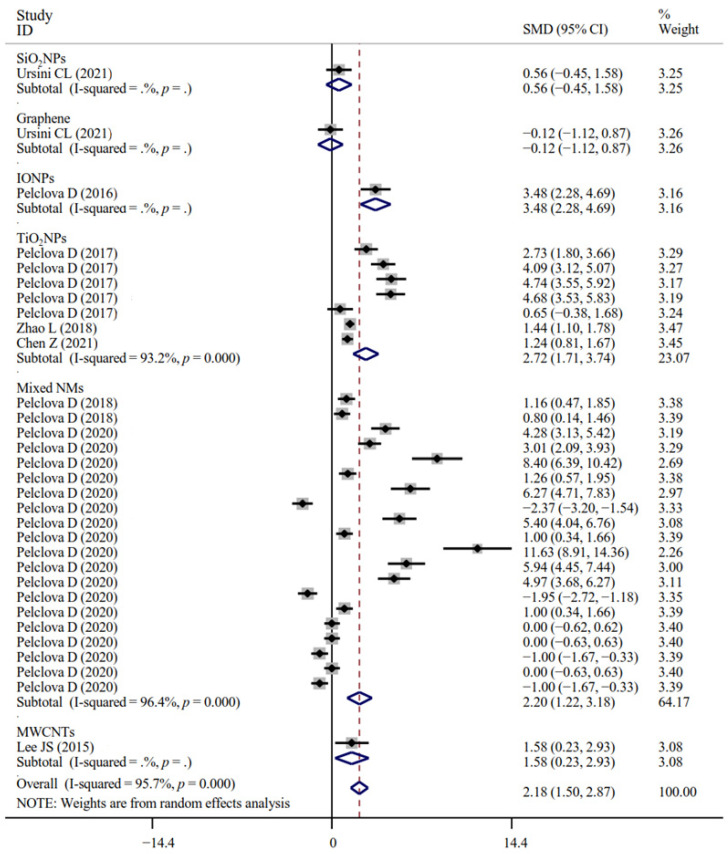
Forest plots assessing the effects of exposure to different NMs on the level of MDA compared with the non-exposed control group. NMs, nanomaterials; SiO_2_NPs, silica oxide nanoparticles; IONPs, iron oxide nanoparticles; TiO_2_NPs, titanium dioxide nanoparticles; MWCNTs, multi-walled carbon nanotubes; MDA, malonaldehyde; SMD, standardized mean difference; CI, confidence interval [15,18,19,31,33,34,43,45].

**Figure 3:**
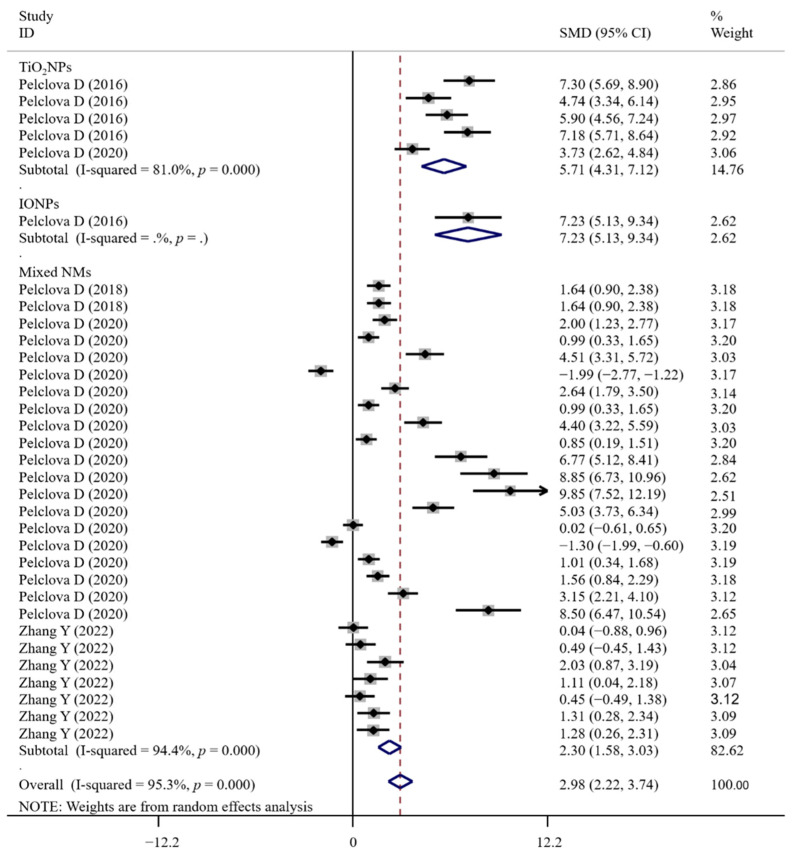
Forest plots assessing the effects of exposure to different NMs on the level of 8-OHG compared with the non-exposed control group. NMs, nanomaterials; IONPs, iron oxide nanoparticles; TiO_2_NPs, titanium dioxide nanoparticles; 8-OHG, 8-hydroxyguanine; SMD, standardized mean difference; CI, confidence interval [15,29,33,43,44].

**Figure 4:**
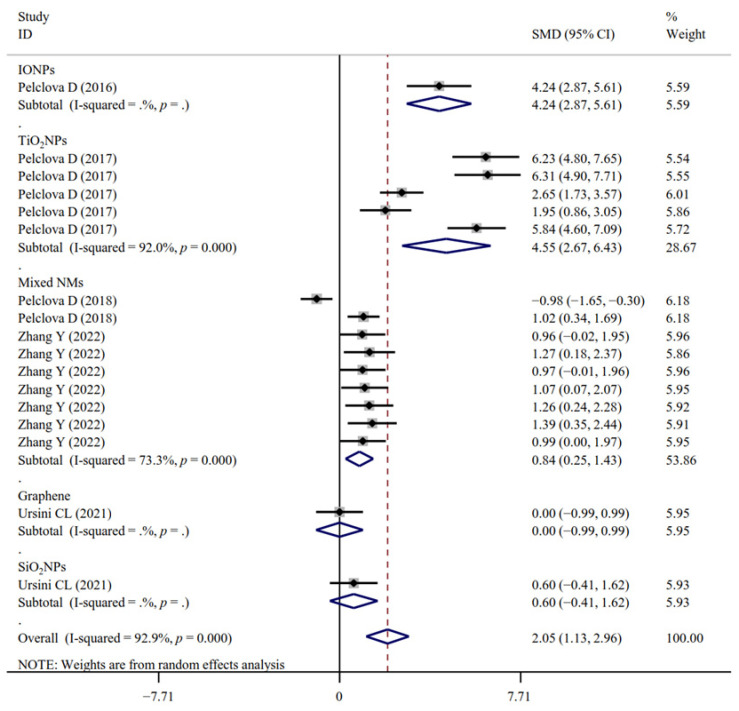
Forest plots assessing the effects of exposure to different NMs on the level of HNE compared with the non-exposed control group. NMs, nanomaterials; SiO_2_NPs, silica oxide nanoparticles; IONPs, iron oxide nanoparticles; TiO_2_NPs, titanium dioxide nanoparticles; HNE, 4-hydroxy-2-nonhenal; SMD, standardized mean difference; CI, confidence interval [15,18,29,31,33,34].

**Figure 5:**
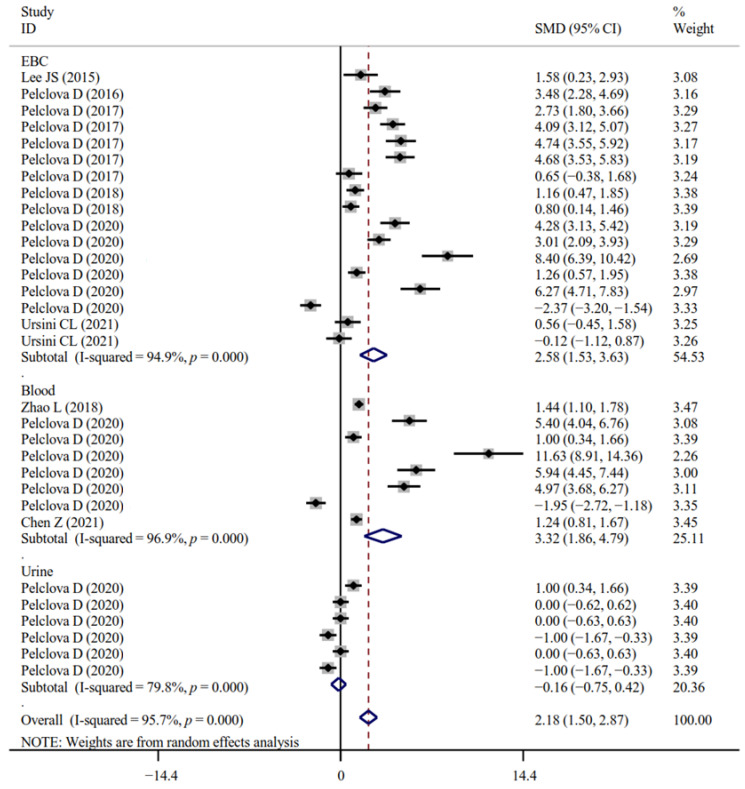
Forest plots assessing the effects of NM exposure on the level of MDA in different samples compared with the non-exposed control group. NMs, nanomaterials; EBC, exhaled breath condensate; MDA, malonaldehyde; SMD, standardized mean difference; CI, confidence interval [15,18,19,31,33,34,41,43,45].

**Figure 6:**
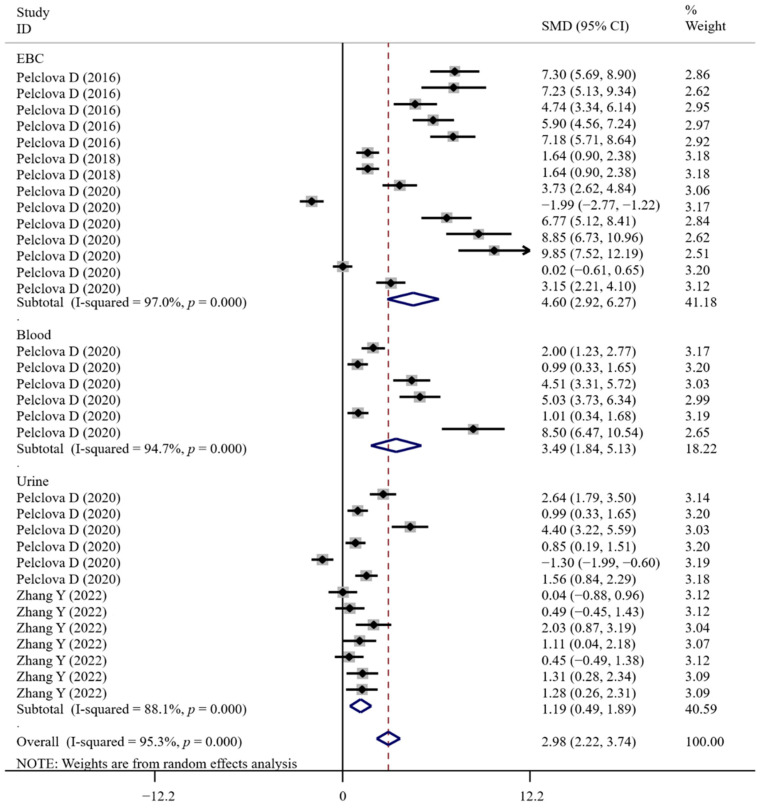
Forest plots assessing the effects of NM exposure on the level of 8-OHG in different samples compared with the non-exposed control group. NMs, nanomaterials; EBC, exhaled breath condensate; 8-OHG, 8-hydroxyguanine; SMD, standardized mean difference; CI, confidence interval [15,29,33,43,44].

**Figure 7:**
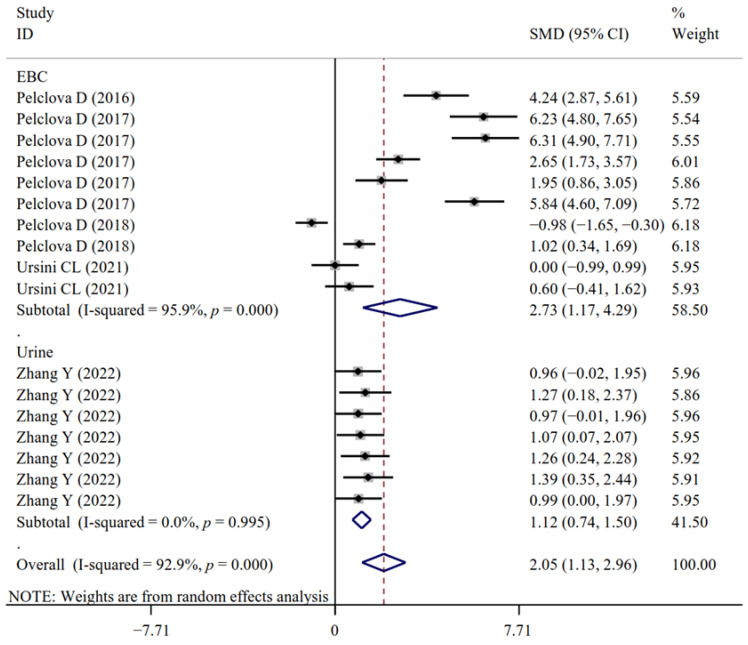
Forest plots assessing the effects of exposure to different NMs on the level of HNE in different samples compared with the non-exposed control group. NMs, nanomaterials; EBC, exhaled breath condensate; HNE, 4-hydroxy-2-nonhenal; SMD, standardized mean difference; CI, confidence interval [15,18,29,31,33,34].

**Figure 8:**
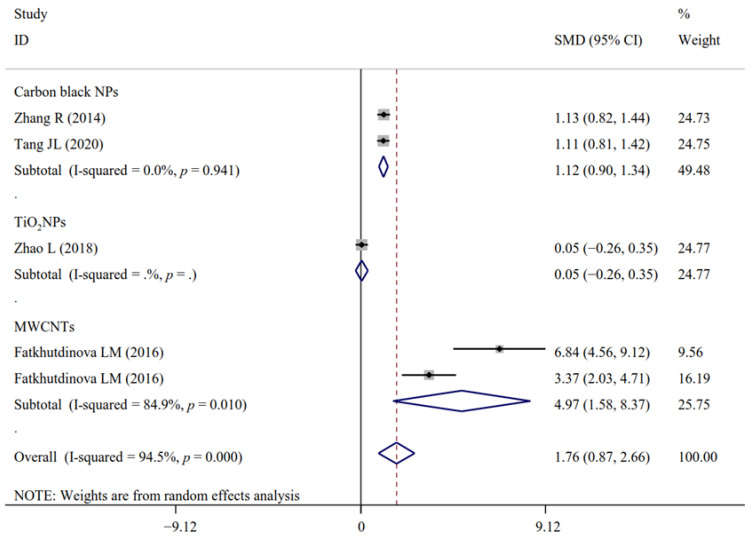
Forest plots assessing the effects of exposure to different NMs on the level of IL-1β compared with the non-exposed control group. NMs, nanomaterials; NPs, nanoparticles; TiO_2_NPs, titanium dioxide nanoparticles; MWCNTs, multi-walled carbon nanotubes; IL-1β, interleukin-1β; SMD, standardized mean difference; CI, confidence interval [17,19,39,40].

The associations between occupational NM exposure and oxidative stress biomarker levels (MDA, Figure 2; GPx; 8-OHG, Figure 3; HNE, Figure 4; 8-Isprostane; 8-isoPGF2a; 3-ClTyr; 5-OHMeU; o-Tyr; 3-NOTyr; aldehydes C6-12) were still significant in most subgroups (with at least two datasets analyzed) stratified by NM types (Table S2). Relative to urinary samples (*p* > 0.05), the levels of MDA (Figure 5), 8-isoPGF2a, 5-OHMeU, o-Tyr, 3-NOTyr and aldehydes C6-12 in EBC and blood samples were more significantly changed by NM exposure. 8-OHG (Figure 6) was found to be significantly increased in EBC, blood and urinary samples; HNE (Figure 7) and 8-Isprostane were significantly increased in EBC and urinary samples. However, the SMD of these three indicators in urinary samples (around 1) was smaller than that obtained in EBC and blood samples (>2) (Table S2). These findings suggest oxidative stress biomarkers in EBC and blood samples, particularly, should be monitored for NM exposed populations.

#### 3.3.2. Association between Occupational NM Exposure and Inflammatory Biomarker Levels

A total of 17, 5, 4, 4, 20, 14, 11, 4, 15, 2, 2, 28, 14, 18, 21, 7, 14, 15, 21, 7 and 3 experimental datasets respectively measured the levels of inflammatory biomarkers FENO, IL-1β, IL-4, IL-5, IL-6, IL-6sR, IL-8, IL-10, TNF-α, MIP-1β, MCP-1, NF-κB, MPO, CRP, CC16, SP-A/D, fibrinogen, VCAM, ICAM, LT-B-E4 and neutrophils in NM-exposed and non-exposed populations (Table 2). The results of meta-analysis revealed that in comparison to the controls, significant increases in FENO (SMD = 0.48; 95% CI, 0.17–0.78; *p* = 0.002), IL-1β (SMD = 1.76; 95% CI, 0.87–2.66; *p* < 0.001), IL-4 (SMD = 2.19; 95% CI, 0.28–4.09; *p* = 0.024), TNF-α (SMD = 1.52; 95% CI, 1.03–2.01; *p* < 0.001), MIP-1β (SMD = 1.61; 95% CI, 0.83–2.38; *p* < 0.001), MPO (SMD = 0.25; 95% CI, 0.16–0.34; *p* < 0.001), fibrinogen (SMD = 0.11; 95% CI, 0.02–0.21; *p* = 0.016), ICAM (SMD = 0.32; 95% CI, 0.14–0.50; *p* < 0.001), LT-B4 (SMD = 2.09; 95% CI, 0.72–3.46; *p* = 0.003), LT-E4 (SMD = 1.65; 95% CI, 0.22–3.07; *p* = 0.024) and significant decreases of IL-6sR (SMD = −0.18; 95% CI, −0.28–−0.09; *p* < 0.001) and MCP-1 (SMD = −0.25; 95% CI, −0.45–−0.04; *p* = 0.018) were found for NM-handling workers. The levels of IL-5, IL-6, IL-8, IL-10, NF-κB, CRP, CC16, SP-A/D, VCAM, LT-C4, LT-D4 and neutrophils were not significantly changed after NM exposure (Table 2).

**Figure 9:**
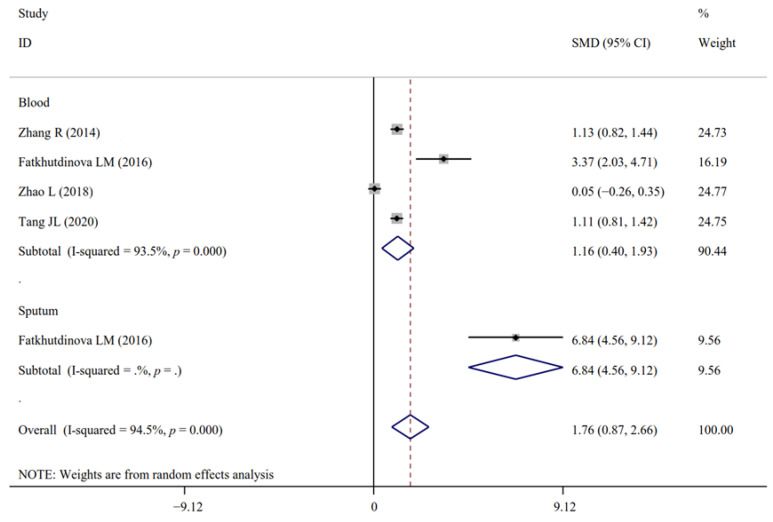
Forest plots assessing the effects of NM exposure on the level of IL-1β in different samples compared with the non-exposed control group. NMs, nanomaterials; IL-1β, interleukin-1β; SMD, standardized mean difference; CI, confidence interval [17,19,39,40].

**Figure 10:**
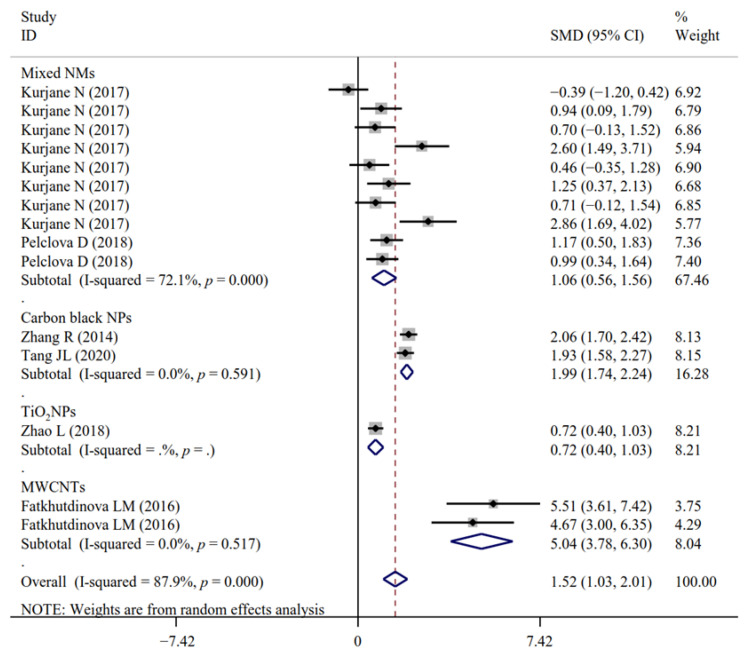
Forest plots assessing the effects of exposure to different NMs on the level of TNF-α compared with the non-exposed control group. NMs, nanomaterials; NPs, nanoparticles; TiO_2_NPs, titanium dioxide nanoparticles; MWCNTs, multi-walled carbon nanotubes; TNF-α, tumor necrosis factor-α; SMD, standardized mean difference; CI, confidence interval [17,19,25,36,39,40].

**Figure 11:**
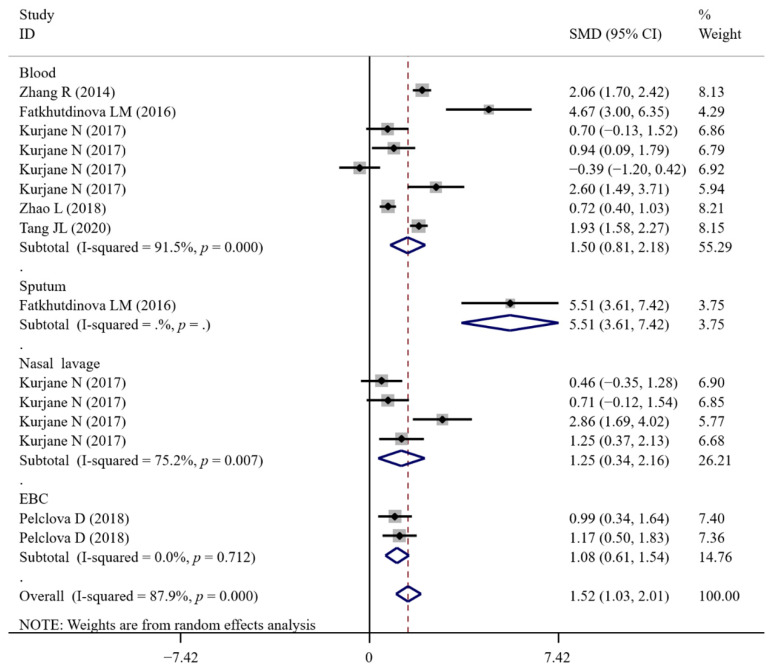
Forest plots assessing the effects of NM exposure on the level of TNF-α in different samples compared with the non-exposed control group. NMs, nanomaterials; EBC, exhaled breath condensate; TNF-α, tumor necrosis factor-α; SMD, standardized mean difference; CI, confidence interval [17,19,25,36,39,40].

Only the effects of NM exposure on the levels of IL-1β (Figure 8 and Figure 9), TNF-α (Figure 10 and Figure 11) and ICAM were still significant in the analyses of subgroups (regardless of NM types and sample sources) with at least two datasets (Table S3). LT-B4 and LT-E4 in the EBC samples (but not urine) were found to be significantly increased by NM exposure. FENO was only observed to be significantly higher in the TiO_2_NP-exposed workers compared with the non-exposed controls (Table S3). NM types (mixed) and sample sources (serum) were the same in all datasets for the analysis of MPO, fibrinogen and IL-6sR. The number of datasets (only two) was small for MIP-1β and MCP-1. Thus, the subgroup analysis was not performed for them.

### 3.4. Publication Bias and Sensitivity Analysis

**Figure 12:**
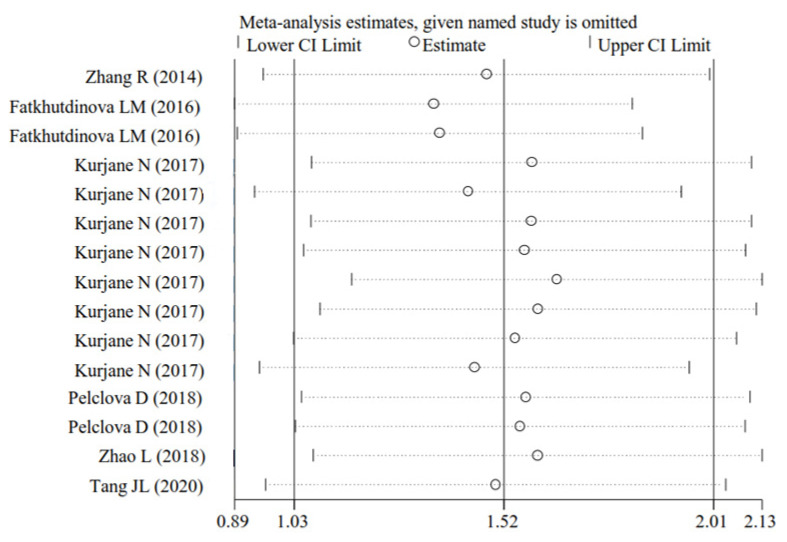
Sensitivity analysis for TNF-α. The vertical axis shows the omitted study. The horizontal axis represents the corresponding pooled estimate when the study is excluded. Every circle indicates the pooled SMD (all of them are close to 1.52 obtained in the overall meta-analysis). The two ends of every broken line represent the respective 95% CI. TNF-α, tumor necrosis factor-α; SMD, standardized mean difference; CI, confidence interval [17,19,25,36,39,40].

Egger’s test showed the publication bias was present for analyses of MDA (*p* = 0.002), HNE (*p* < 0.001), HHE (*p* = 0.001), 8-Isprostane (*p* < 0.001), 8-isoPGF2a (*p* < 0.001), 8-OHdG (*p* < 0.001), 8-OHG (*p* < 0.001), 3-ClTyr (*p* < 0.001), 5-OHMeU (*p* < 0.001), o-Tyr (*p* < 0.001), 3-NOTyr (*p* < 0.001), aldehyde C6 (*p* = 0.009), aldehyde C11 (*p* = 0.003), total aldehydes C6-C12 (*p* < 0.001), FENO (*p* < 0.001), IL-4 (*p* = 0.001), IL-5 (*p* < 0.001), ICAM (*p* = 0.007), LT-B4 (*p* = 0.005), LT-C4 (*p* < 0.001), LT-D4 (*p* = 0.004), LT-E4 (*p* = 0.001) and neutrophils (*p* = 0.015) (Table 2). Therefore, the trim-and-fill method was then conducted. After missing studies were imputed, the adjusted result still showed that the levels of MDA (SMD = 0.88; 95% CI, 0.13–1.62; *p* = 0.001), HNE (SMD = 1.75; 95% CI, 0.79–2.72; *p* < 0.001), 8-OHdG (SMD = 0.32; 95% CI, 0.07–0.57; *p* = 0.011), 8-OHG (SMD = 3.55; 95% CI, 1.53–8.23; *p* = 0.003), 5-OHMeU (SMD = 0.88; 95% CI, 0.15–1.61; *p* = 0.018), o-Tyr (SMD = 0.94; 95% CI, 0.29–1.58; *p* = 0.005), 3-NOTyr (SMD = 1.14; 95% CI, 0.22–2.07; *p* = 0.016), aldehyde C6 (SMD = 3.87; 95% CI, 1.49–6.26; *p* = 0.001), aldehyde C11 (SMD = 1.85; 95% CI, 0.66–3.05; *p* = 0.001) and total aldehydes C6-C12 (SMD = 1.73; 95% CI, 1.06–2.41; *p* < 0.001) were significantly increased after NM exposure. The effects on the levels of HHE (*p* = 0.568), 8-Isprostane (*p* = 0.083), 8-isoPGF2a (*p* = 0.236), 3-ClTyr (*p* = 0.064), FENO (*p* = 0.997), IL-4 (*p* = 0.384), ICAM (*p* = 0.094), LT-B4 (*p* = 0.860) and LT-E4 (*p* = 0.787) were no longer significant after correction. The negative effects on the levels of IL-5, LT-C4, LT-D4 and neutrophils were maintained after correction. Sensitivity analyses showed that pooled estimates remained in the same directions when the studies were omitted one by one, suggesting the stability and reliability of this meta-analysis and the results were not influenced by any one study (Figure 12).

## 4. Discussion

There have been studies stating the potential relationships of oxidative stress biomarkers [47,48] and FENO [49] with occupational exposure to NMs, but all of them only reviewed the results of known individual articles. No meta-analyses have been conducted to synthesize all data from each study to overcome the low statistical power and achieve a comprehensive and reliable conclusion. In the present study, we, for the first time, included 28 epidemiological studies with 2636 participants and performed a meta-analysis to examine the effects of NM exposure on oxidative stress and inflammatory biomarkers. After overall analysis, subgroup meta-analyses, trim-and-fill adjusted estimates and the sensitivity analysis, we found occupational NM exposure was significantly associated with increases in the levels of MDA, HNE, aldehydes C6-12, 8-OHG, 5-OHMeU, o-Tyr, 3-NOTyr, IL-1β, TNF-α, MPO, fibrinogen, and decreases in the levels of GPx and IL-6sR.

Existing evidence from in vitro and in vivo studies supported that NM inhalation exposure can induce excessive production of reactive oxygen (ROS, including super oxides, superoxide radicals, hydroxyl radicals and hydrogen peroxide) and reactive nitrogen species (RNS, including peroxynitrite anion and nitric oxide) [50,51]. The ROS could react with the chain polyunsaturated fatty acids in the membrane to trigger the process of lipid peroxidation, resulting in the release of reactive, toxic aldehydes, including MDA, HNE and aldehydes C6-C12 [52]. ROS can attack DNA and RNA to induce oxidative modifications on guanine or thymine bases, leading to the generation of 8-OHdG, 5-OHMeU (both were DNA damage biomarkers) and 8-OHG (RNA damage biomarker) [53]. Hydroxyl radicals can oxidize phenylalanine into o-Tyr, and RNS can mediate the nitration of p-tyrosine to form 3-NOTyr [54]. Thus, the increases in these ROS/RNS-related indicators were speculated to be associated with the oxidative stress damages induced by NM exposure. This hypothesis had been demonstrated by some authors. For example, the summary analysis of 49 data by An et al. showed that the level of MDA in model rats or mice was increased by 5.52-fold after TiO_2_NP exposure compared with the controls [12]. A short-time exposure of silver NPs was reported to induce the formation of 4-HNE-protein adducts in SUM159 cells to drive cell death [55]. Compared to non-exposed cells, cells exposed to palladium NPs were observed to have an increased accumulation of nuclear acid damage biomarkers, especially 8-OHG (the level of which seemed to be higher than that of 8-OHdG, indicating RNA damage may be more severe) [56]. Consistent with these model studies, we also identified that the levels of MDA, HNE and 8-OHG were significantly increased in occupationally NM-exposed workers. Also, the increase fold of 8-OHG (SMD = 2.98) was higher than 8-OHdG (SMD = 1, which was even non-significant in the subgroup analysis) and 5-OHMeU (SMD = 1.9). Aldehydes C6-C12, 3-NOTyr and o-Tyr were only measured in NM-exposed workers [15,16,29,44], not in cell and animal studies. However, in line with the predicted theory, our meta-analysis also confirmed positive correlations of these three biomarkers with NM exposure. GPx is an enzymatic free radical scavenger that could protect the body from ROS-induced damages. The accumulation of ROS and RNS after NM exposure was attributed to a reduced level and inactivation of GPx [57,58]. Similar to these cell studies, our meta-analysis identified a disturbance of GPx in NM-exposed workers.

Other than oxidative stress, activation of inflammation is one of the key mechanisms involved in NM-related hazardous effects [13]. NM-induced inflammatory response, on one hand, may be a result mediated by excessive ROS via activation of the mitogen-activated protein kinase-NF-κB signaling pathway [59,60]; on the other hand, it may be associated with activation of ROS-independent hypoxia-inducible factor signaling pathways [61]. Main biomarkers reflecting the inflammatory response are various cytokines released by immune cells, such as IL-1β, TNF-α and IL-6sR. It has been reported that cells or rats exposed to NMs exhibited upregulations of IL-1β and TNF-α; inhibitions of these two biomarkers reversed NM exposure-induced cell death and pathological changes in tissues [62,63]. In agreement with these studies, we also detected statistically significant increased levels of IL-1β and TNF-α in NM-exposed employees. IL-6sR was proved to bind with soluble gp130 to form a complex that inhibited IL-6 trans-signaling-mediated pro-inflammatory effects [64]. The level of IL-6sR was shown to be lower in inflammatory diabetes patients compared with healthy subjects [65]. Theoretically, IL-6sR was downregulated in NM-exposed workers, which was confirmed in our meta-analysis. Although MPO and fibrinogen are not cytokines, accumulated studies have demonstrated their links with inflammation-related diseases, including NM exposure [66,67]. MPO, a heme enzyme expressed in neutrophils, monocytes and macrophages, was suggested to drive inflammation due to its roles in catalyzing the oxidation reaction to generate ROS [68]. Fibrinogen was shown to directly stimulate the production of cytokines by the mitogen-activated protein kinase-NF-κB signaling pathway [69]. Similar to these findings, we also verified the levels of MPO and fibrinogen were significantly higher in NM-exposed workers than those in non-exposed populations.

The oxidative stress and inflammatory biomarkers were measured in multiple specimen types, including EBC, blood, urine, sputum and nasal lavage. Subgroup analysis indicated relative to urinalysis (negative results or small SMD), oxidative stress biomarkers (MDA, HNE, aldehydes C6-12, 8-OHG, 5-OHMeU, o-Tyr, 3-NOTyr and GPx) in EBC and blood samples were significantly or strongly changed by NM exposure. IL-1β, TNF-α, MPO, fibrinogen and IL-6sR were all detected in EBC, blood, sputum and nasal lavage samples and their results were all significant. These findings reflected the fact that NMs may enter into the respiratory tract, blood, other inner organs and urine successively after long-term exposure [70]. The NMs may be deposited in the human body, but not cleared and then released into the urine, or eliminated mainly by mucociliary clearance and ingested [71], ultimately contributing to slight or non-significant changes of these indicators in the urine samples. Furthermore, the negative results of oxidative stress biomarkers in urinary samples may also be associated with the following reasons: (1) it has been reported that there is a time window to detect the responses of urinary biomarkers after exposure. If the sample is not collected in the sensitive time windows, the indicators may be seldomly changed after exposure [29,72]. For example, Zhang et al. detected slower generation and/or urinary excretion kinetics of urinary 8-OHG and HNE in NM- exposed workers. These two biomarkers were only significantly increased at 36 h (an acute model) or three weeks (a chronic model) post-exposure. o-Tyr and 5-OHMeU were not statistically elevated in the longest sampling time points [29]; (2) the elimination half-lives of oxidative stress biomarkers in plasma were also found to be longer than that in urine [73], which may result in their low levels in urinary samples even if the same sampling time points were set as the blood samples; (3) some oxidative stress biomarkers (e.g., HNE and other aldehydes) are intermediary oxidation products which can be fed by a precursor/parent molecule, but consumed by subsequent oxidation reactions or adduct formation. Hereby, the overall concentrations for themselves in urine may be not high; and (4) liquid chromatography electrospray ionization—mass spectrometry/mass spectrometry was used for analyses of urinary oxidative stress biomarkers in all included studies. Although being sensitive relative to the enzyme-linked immunosorbent assay [74,75], it may be still not be the optimal technique for analysis of urinary samples and more new generations of mass spectrometers [76,77] should be applied to further confirm the results in urinary samples to avoid technique-derived deviations. More interestingly, there were published literatures that suggested urinary oxidative stress indicators were excellent biomarkers to identify persons exposed to toxic elements (diagnostic accuracy of 3-NOTyr = 0.753) [78] and predict the hazard effects [79]. The levels of urinary and circulating oxidative stress markers (e.g., 8-OHdG [32], MDA [80]) in occupationally exposed workers were also observed to be significantly correlated. Thus, the biomarker roles of oxidative stress indicators in urine samples should not be discounted and need to be confirmed by designing better experimental protocols in the future (including considering the sampling time windows and the analytical methods).

Several limitations should be addressed. First, the number of available publications and the sample size in each study were small; thus, the results of some indicators may be still inconclusive (such as MCP-1 and MIP-1β in overall analysis as well as FENO in the subgroup analysis). Second, this meta-analysis only preliminarily estimated the association of NM exposure with the oxidative stress and inflammatory biomarkers. The specific threshold values that distinguish NM-exposed populations and normal controls (or pre- and post-exposure) and predict the poor outcomes remain unclear for these biomarkers [78,81]. Which one (or which combination) [82] is the optimal biomarker remains under-investigated. These two issues need to be resolved by the receiver operating characteristic curve analysis and multivariate regression analysis [78,79,81,82]. The reference ranges of significant oxidative stress and inflammatory biomarkers we identified also should be calculated to better explain the biomarker roles of them in different disease settings [74,75,83,84]. Third, meta-analysis was performed based on the mean and SD data of exposed and non-exposed groups collected from each study, not the adjusted results for potential confounders (such as sex, age, smoking or drinking) because they were only provided by some studies and the confounders were different across the studies. Fourth, we excluded the self-control studies (that is, comparisons between before and after exposure) because the exposure time was relatively short (only some hours, which may be meaningless for assessment of long-term exposure effects) and the indicators were different among them (leading to only few results that could be combined). Accordingly, to further confirm biomonitoring effects of our identified biomarkers, more studies that prospectively include workers (just into the factory, previously not exposed to NMs) and assign them to exposed and non-exposed workplaces followed by detecting the levels of biomarkers in multiple follow-up time points are needed.

## 5. Conclusions

Our meta-analysis suggests that oxidative stress indicators (MDA, HNE, aldehydes C6-12, 8-OHG, 5-OHMeU, o-Tyr, 3-NOTyr and GPx) in EBC and blood samples, as well as inflammatory mediators (IL-1β, TNF-α, MPO, fibrinogen and IL-6sR) in EBC, blood, sputum and nasal lavage samples were significantly associated with NM exposure. They may represent potential biomarkers for the biological monitoring of the population exposed to NMs at the workplace. The biomarker roles of oxidative stress indicators in urinary samples need to be confirmed by designing experiments with different sampling time points and new analytical methods.

## Figures and Tables

**Table 1:** Characteristics of included studies.

Studies	Year	Countries	Sample Size (Exposed/Unexposed)	Study Design	Subjects		NM Type	Specimens	Outcome Measured	NOS
					Exposed	Unexposed				
Zhang Y [29]	2022	USA	15/20	Cross-sectional	Volunteer spending two or three days (5–6 h/day) in a copy center; full-time copier operators for over two years	Volunteers spending an equal amount of time in an office; workers not involved with any printing and photocopying activities	Mixed NMs	Urine	HNE, 8-Isprostane, 8-OHdG, 8-OHG, 5-OHMeU, o-Tyr	9
Ursini CL [18]	2021	Italy	12/11	Cross-sectional	Workers in a research laboratory producing NMs for >three weeks	Workers in the administrative offices	Graphene; SiO_2_NPs	EBC, EB, urine, blood	MDA, HNE, 8-Isprostane, 8-OHdG, FENO, IL-6, IL-8	9
Wu WT [14]	2021	China (Taiwan)	80/69	Cross-sectional	Workers in NM manufacturing and/or handling factories for 3.2 ± 2.4 years	Office workers who never entered the production or manufacturing area and did not handle NMs	CNTs; SiO_2_NPs; TiO_2_NPs	EBC, urine	8-isoPGF2a, 8-Isprostane	8
Chen Z [45]	2021	China (mainland)	56/44	Cross-sectional	Production workers in NM manufacturing plants for >one year	Workers from management positions of the same plant	TiO_2_NPs	Blood	MDA, SOD	7
Pelclova D [43]	2020	Czech	20/20; 21/18	Cross-sectional	Workers at the NM production plants for 12.2 ± 9.3 (2017) or 13.9 ± 9.4 (2018) years	Workers from the same plant, but not employed in dusty workplaces	Mixed NMs	EBC, blood, urine	MDA, 8-isoPGF2, 8-OHdG, 8-OHG, 5-OHMeU, o-Tyr, 3-NOTyr	9
Yu M [38]	2020	China (mainland)	23/23	Cross-sectional	Workers in a plant that manufactures ferric NMs for 2 (0.5–2.5) years	Workers from another plant who did not handle and/or produce NMs	IONPs	Blood	8-OHdG	9
Tang JL [39]	2020	China (mainland)	85/106	Cross-sectional	Workers who have bagged newly manufactured NMs for more than 6 months	Workers from a local water authority with no specific exposure to NMs	Carbon black NPs	Blood	IL-6, TNF-α, IL-1β, IL-8, MIP-1β, MCP-1, CRP	9
Wu WT [27]	2019	China (Taiwan)	206/108	Panel	Workers in NM manufacturing and/or using plants for 8–11 years	Workers at the same plants, but not handle NMs	Mixed NMs	Blood, EBC, urine	FENO, CC16, NF-κB, 8-OHdG, 8-isoPGF2, SOD, GPx, CRP, IL-6, IL-6sR, MPO, fibrinogen, VCAM, ICAM	8
Zhao L [19]	2018	China (mainland)	83/85	Cross-sectional	Workers in NM manufacturing plant for average 5 (4–9.25) years	Workers from the same plant without occupational exposure to NMs	TiO_2_NPs	Blood	IL-6, IL-8, TNF-α, IL-1β, IL-10, CRP, MDA, SOD, CC16, SP-A, SP-D, VCAM, ICAM	8
Pelclova D [33]	2018	Czech	19/19	Cross-sectional	NM-synthesizing and processing researchers for average 18.0 ± 10.3 years	Workers not employed in this plant, nor occupationally exposed to NMs	Mixed NMs	EBC	MDA, HHE, HNE, 8-Isprostane, 8-OHdG, 8-OHG, 5-OHMeU, o-Tyr, 3-NOTyr, 3-ClTyr	9
Pelclova D [36]	2018	Czech	20/21	Cross-sectional	NMs researchers for 17.8 ± 10.0 years	Office workers in the same town	Mixed NMs	EBC	FENO, LT-B4, LT-C4, LT-D4, LT-E4, TNF-α, IL-4, IL-10, IL-5	9
Kuijpers E [42]	2018	Netherlands	22/42; 13/6	Cross-sectional	Workers of a company commercially producing NMs	Workers at the same company but did not produce or handle NMs, or from neighboring industries	MWCNTs	Blood	ICAM	8
Kurjane N [25]	2017	Latvia	24/12	Cross-sectional	Workers in metalworking or woodworking company	Office workers	Mixed NMs	Blood, nasal lavage	IL-8, TNF-α	8
Vlaanderen J [26]	2017	Netherlands	22/39	Cross-sectional	Workers of an NM-producing facility	Workers in a department of the same facility, but did not produce or use NM, or in neighboring facilities	MWCNTs	Blood	CC16, SP-A, SP-D	9
Glass DC [30]	2017	Australia	34/55	Panel	Workers in university research laboratories where NMs were handled	Offices workers in the same laboratories, but no NMs handled	Mixed NMs	Blood	FENO, CRP, neutrophils	8
Pelclova D [31]	2017	Czech	22/14	Cross-sectional	Office employees (who visited for a daily average of 0.23 ± 0.15 h the production workshops) of a NM producing facility for 15.5 ± 3.6 years	Workers not employed in the factory	TiO_2_NPs	EBC	MDA, HHE, HNE, 8-Isprostane, aldehydes C6-C12	8
Liou SH [32]	2017	China (Taiwan)	87/43	Cross-sectional	Workers in NM manufacturing and/or handling factories for average 2.69 years	Workers non-exposed to NMs	TiO_2_NPs; SiO_2_NPs; ITONPs	EBC, urine, blood	8-OHdG, 8-isoprostane	8
Pelclova D [34]	2017	Czech	34/45	cross-sectional	Production workers or worker in research wing of the factory for 3.8–9.7 years	Workers not occupationally exposed to NMs	TiO_2_NPs	EBC	MDA, HNE, HHE, 8-Isprostane, aldehydes C6- C12	7
Fireman E [46]	2017	Israel	25/35	Cross-sectional	Workers exposed to occupational NMs from industrial sources for 26.36 ± 15.86 years	Workers not occupationally exposed to any NMs	Mixed NMs	Sputum	Neutrophils	7
Pelclova D [15]	2016	Czech	14/14	Cross-sectional	Workers of an NM-producing facility for 10 ± 4 years	Workers not employed in related factory	IONPs	EBC	MDA, HHE, HNE, 8-isoPGF2, 8-OHdG, 8-OHG, 5-OHMeU, o-Tyr, 3-ClTyr, 3-NOTyr, aldehydes C6-C12	8
Pelclova D [28]	2016	Czech	30/67	Cross-sectional	Workers and office employees (who also visited the production workshops for a daily average of 0.23 ± 0.15 h) of an NM-producing facility for average 8.93%#x2012;15.45 years	Workers not employed in the factory	TiO_2_NPs	EBC, urine	FENO, LT-B4, LT-C4, LT-D4, LT-E4	8
Pelclova D [16]	2016	Czech	22/14	Cross-sectional	Office employees (who visited for a daily average of 0.23 ± 0.15 h the production workshops) of a TiO_2_NPs producing facility for 15.5 ± 3.6 years	Workers not employed in the factory	TiO_2_NPs	EBC	8-OHdG, 8-OHG, 5-OHMeU, o-Tyr, 3-NOTyr, 3-ClTyr	7
Liou SH [35]	2016	China (Taiwan)	127/100	Cross-sectional	Workers in NM manufacturing and/or handling factories for average 2.60 ± 2.23 years	Workers non-exposed to NMs	TiO_2_NPs; SiO_2_NPs; ITONPs	Urine, blood	8-OHdG, SOD, GPx	7
Fatkhutdinova LM [40]	2016	Russia	10/12	Cross-sectional	Workers in contact with MWCNT aerosol for more than one year	Workers not exposed to MWCNT aerosol	MWCNTs	Blood, sputum	IL-6, IL-8, TNF-α, IL-1β, IL-4, IL-5, IL-10	8
Pelclova D [44]	2016	Czech	34/45	Cross-sectional	Production workers or worker in research wing of the factory for 3.8–9.7 years	Workers not occupationally exposed to NMs	TiO_2_NPs	EBC	8-OHdG, 8-OHG, 5-OHMeU, o-Tyr, 3-NOTyr, 3-ClTyr, aldehydes C6-C12	9
Lee JS [41]	2015	Korea	9/4	Cross-sectional	CNT manufacturing workers for 3.9 ± 3.9 years	Office workers	MWCNTs	EBC	MDA, HHE	9
Zhang R [17]	2014	China (mainland)	81/104	Cross-sectional	Workers packing NPs of carbon black for 12.5 ± 11.07 years	Workers from a water plant	Carbon black NPs	Blood	IL-6, IL-8, TNF-α, IL-1β, MCP-1, MIP-1β	9
Liao HY [37]	2014	China (Taiwan)	124/77	Cross-sectional	NM-handling workers for 3.22 years	Workers at the same factories, but did not handle NMs	Mixed NMs	Blood, urine	CC16, NF-κB, 8-OHdG, 8-Isprostane, SOD, GPx, CRP, IL-6, IL-6sR, MPO, fibrinogen, VCAM, ICAM	8

NMs, nanomaterials; NPs, nanoparticles; SiO_2_NPs, silica oxide nanoparticles; IONPs, iron oxide nanoparticles; TiO_2_NPs, titanium dioxide nanoparticles; ITONPs, indium tin oxide nanoparticles; MWCNTs, multi-walled carbon nanotubes; EBC: exhaled breath condensate; EB, exhaled breath; MDA, malonaldehyde; HNE, 4-hydroxy-2-nonhenal; 8-OHdG, 8- hydroxydeoxyguanosine; 8-OHG, 8-hydroxyguanine; FENO, fraction of exhaled nitric oxide; CC16, club cell secretory protein 16; SP-A, surfactant protein A; SP-D, surfactant protein D; IL, interleukin; TNF, tumor necrosis factor; HHE, 4-hydroxy-trans-hexenale; 8-isoPGF2a, 8-isoProstaglandin F2α; 5-OHMeU, 5-hydroxymethyl uracil; o-Tyr, o-tyrosine; 3-ClTyr, 3-chlorotyrosine; 3-NOTyr, 3-nitrotyrosine; SOD, superoxide dismutase; GPx, glutathione peroxidase; VCAM, vascular cell adhesion molecule; ICAM, intercellular adhesion molecule; CRP, C-reactive protein; MPO, myeloperoxidase; IL-6sR, IL-6 soluble receptor; LT-B4, leukotriene B4; LT-C4, leukotriene C4; LT-D4, leukotriene D4; LT-E4, leukotriene E4; MCP-1, monocyte chemoattractant protein-1; MIP-1β, macrophage inflammatory protein-1β; NF-κB, nuclear factor kappa-B.

**Table 2:** Meta-analysis.

Variables	No.	SMD	95% CI	*p*_A_-Value	I^2^	*p*_H_-Value	Model	Egger *p*
Oxidative stress biomarkers								
MDA	31	2.18	1.50, 2.87	<0.001	95.7	<0.001	R	0.002
SOD	19	−0.24	−0.44, −0.03	0.024	84.6	<0.001	R	0.975
GPx	17	−0.31	−0.52, −0.11	0.003	82.8	<0.001	R	0.944
HNE	17	2.05	1.13, 2.96	<0.001	92.9	<0.001	R	<0.001
HHE	9	4.27	2.13, 6.40	<0.001	97.1	<0.001	R	0.001
8-Isprostane	26	1.13	0.76, 1.50	<0.001	89.5	<0.001	R	<0.001
8-isoPGF2a	35	1.22	0.83, 1.60	<0.001	94.5	<0.001	R	<0.001
8-OHdG	75	1.00	0.79, 1.21	<0.001	93.1	<0.001	R	<0.001
8-OHG	33	2.98	2.22, 3.74	<0.001	95.3	<0.001	R	<0.001
3-ClTyr	8	4.36	2.56, 6.16	<0.001	95.6	<0.001	R	<0.001
5-OHMeU	33	1.90	1.23, 2.58	<0.001	94.9	<0.001	R	<0.001
o-Tyr	33	1.81	1.22, 2.41	<0.001	93.7	<0.001	R	<0.001
3-NOTyr	26	2.63	1.74, 3.52	<0.001	96.1	<0.001	R	<0.001
Aldehyde C6	6	5.53	3.29, 7.77	<0.001	93.6	<0.001	R	0.009
Aldehyde C7	6	3.53	1.83, 5.23	<0.001	93.0	<0.001	R	0.213
Aldehyde C8	6	3.46	1.48, 5.45	0.001	94.6	<0.001	R	0.407
Aldehyde C9	6	4.88	2.69, 7.06	<0.001	94.1	<0.001	R	0.081
Aldehyde C10	6	4.80	2.93, 6.66	<0.001	92.5	<0.001	R	0.058
Aldehyde C11	6	2.30	1.16, 3.44	<0.001	90.0	<0.001	R	0.030
Aldehyde C12	6	1.75	0.77, 2.73	<0.001	87.3	<0.001	R	0.949
Aldehydes C6-12	60	3.45	2.80, 4.10	<0.001	95.9	<0.001	R	<0.001
Inflammatory biomarkers								
FENO	17	0.48	0.17, 0.78	0.002	86.9	<0.001	R	<0.001
IL-1β	5	1.76	0.87, 2.66	<0.001	94.5	<0.001	R	0.137
IL-4	4	2.19	0.28, 4.09	0.024	94.1	<0.001	R	0.001
IL-5	4	1.43	−0.02, 2.88	0.053	91.5	<0.001	R	<0.001
IL-6	20	0.31	0.00, 0.63	0.050	92.3	<0.001	R	0.899
IL-6sR	14	−0.18	−0.28, −0.09	<0.001	38.0	0.074	F	0.985
IL-8	11	0.11	−0.48, 0.70	0.715	90.9	<0.001	R	0.282
IL-10	4	0.64	−0.28, 1.56	0.175	88.9	<0.001	R	0.258
TNF-α	15	1.52	1.03, 2.01	<0.001	87.9	<0.001	R	0.541
MIP-1β	2	1.61	0.83, 2.38	<0.001	90.8	0.001	R	-
MCP-1	2	−0.25	−0.45, −0.04	0.018	0.0	0.579	F	-
NF-κB	28	−0.05	−0.15, 0.06	0.389	58.0	<0.001	R	0.632
MPO	14	0.25	0.16, 0.34	<0.001	0.0	0.453	F	0.515
CRP	18	0.13	−0.09, 0.34	0.250	84.8	<0.001	R	0.131
CC16	21	−0.05	−0.13, 0.04	0.281	39.3	0.034	F	0.086
SP-A	7	−0.06	−0.30, 0.19	0.655	0.0	0.763	F	0.794
SP-D	7	0.01	−0.45, 0.47	0.973	51.3	0.055	R	0.911
Fibrinogen	14	0.11	0.02, 0.21	0.016	0.0	0.892	F	0.242
VCAM	15	0.07	−0.02, 0.16	0.107	46.6	0.024	F	0.584
ICAM	21	0.32	0.14, 0.50	<0.001	72.2	<0.001	R	0.007
LT-B4	7	2.09	0.72, 3.46	0.003	96.1	<0.001	R	0.005
LT-C4	7	1.19	−0.05, 2.42	0.061	95.8	<0.001	R	<0.001
LT-D4	7	1.05	−0.14, 2.24	0.083	95.6	<0.001	R	0.004
LT-E4	7	1.65	0.22, 3.07	0.024	96.6	<0.001	R	0.001
Neutrophils	3	0.19	−0.10, 0.48	0.202	0.0	0.535	F	0.015

MDA, malonaldehyde; SOD, superoxide dismutase; GPx, glutathione peroxidase; HNE, 4-hydroxy-2-nonhenal; HHE, 4-hydroxy-trans-hexenale; 8-OHdG, 8-hydroxydeoxyguanosine; 8-OHG, 8-hydroxyguanine; 8-isoPGF2a, 8-isoProstaglandin F2α; 5-OHMeU, 5-hydroxymethyl uracil; o-Tyr, o-tyrosine; 3-ClTyr, 3-chlorotyrosine; 3-NOTyr, 3-nitrotyrosine; FENO, fraction of exhaled nitric oxide; IL, interleukin; IL-6sR, IL-6 soluble receptor; TNF, tumor necrosis factor; MCP-1, monocyte chemoattractant protein-1; MIP-1β, macrophage inflammatory protein-1β; NF-κB, nuclear factor kappa-B; MPO, myeloperoxidase; CRP, C-reactive protein; CC16, club cell secretory protein 16; SP-A, surfactant protein A; SP-D, surfactant protein D; VCAM, vascular cell adhesion molecule; ICAM, intercellular adhesion molecule; LT-B4, leukotriene B4; LT-C4, leukotriene C4; LT-D4, leukotriene D4; LT-E4, leukotriene E4; SMD, standardized mean difference; CI, confidence interval; F, fixed-effects; R, random-effects; *p*_H_-Value, significance for heterogeneity; *p*_A_-Value, significance for associations.

## Data Availability

All data can be obtained in published articles and Table S1.

## Supplementary Materials

The following supporting information can be downloaded at: https://www.mdpi.com/article/10.3390/antiox11112182/s1, Table S1: Extracted data for the meta-analysis, Table S2: Subgroup analysis for oxidative stress biomarkers, Table S3: Subgroup analysis for inflammatory biomarkers.
