# Supplementary material for: Oxidative Stress and Inflammatory Biomarkers for Populations with Occupational Exposure to Nanomaterials: A Systematic Review and Meta-Analysis
Source: Antioxidants (Basel). 2022 Nov 4;11(11):2182. doi: 10.3390/antiox11112182 (PMC9687069; doi:10.3390/antiox11112182)
Supplement: Supplementary file 1 [file antioxidants-11-02182-s001.zip › Table S2.pdf]

Table S2. Subgroup analysis for oxidative stress biomarkers

Variables			No.	SMD	95%CI	p_A-Value	I^2	p_H-Value	Model
MDA	NM	SiO_2NPs	1	0.56	−0.45,1.58	0.279	-	-	R
	type	Graphene	1	−0.12	−1.12,0.88	0.812	-	-	R
		IONPs	1	3.48	2.28,4.69	<0.001	-	-	R
		TiO_2NPs	7	2.72	1.71,3.74	<0.001	93.2	<0.001	R
		Mixed NMs	20	2.20	1.22,3.18	<0.001	96.4	<0.001	R
		MWCNTs	1	1.58	0.23,2.93	0.022	-	-	R
	Sample	EBC	17	2.58	1.53,3.63	<0.001	94.9	<0.001	R
	source	Blood	8	3.33	1.86,4.79	<0.001	96.9	<0.001	R
		Urine	6	−0.16	−0.75,0.42	0.584	79.8	<0.001	R
SOD	NM	Mixed NMs	14	−0.06	−0.22,0.10	0.436	66.3	<0.001	R
	type	TiO_2NPs	3	−0.59	−1.01,−0.18	0.005	73.4	0.023	R
		SiO_2NPs	1	−0.48	−0.85,−0.10	0.012	-	-	R
		ITONPs	1	−1.45	−1.82,−1.09	<0.001	-	-	R
GPx	NM	SiO_2NPs	1	−0.90	−1.29,−0.52	<0.001	-	-	R
	type	ITONPs	1	−1.37	−1.73,−1.01	<0.001	-	-	R
		TiO_2NPs	1	−0.85	−1.26,−0.43	<0.001	-	-	R
		Mixed NMs	14	−0.17	−0.33,−0.01	0.035	65.1	<0.001	R
HNE	NM	SiO_2NPs	1	0.60	−0.42,1.62	0.246	-	-	R
	type	Graphene	1	0.00	−1.00,1.00	1.000	-	-	R
		IONPs	1	4.24	2.87,5.61	<0.001	-	-	R
		TiO_2NPs	5	4.55	2.67,6.43	<0.001	92.0	<0.001	R
		Mixed NMs	9	0.84	0.25,1.43	0.005	73.3	<0.001	R
	Sample	EBC	10	2.73	1.17,4.29	0.001	95.9	<0.001	R
	source	Urine	7	1.12	0.74,1.51	<0.001	0.0	0.995	F
HHE	NM	IONPs	1	7.91	5.64,0.19	<0.001	-	-	R
	type	TiO_2NPs	5	5.99	2.30,9.69	0.001	97.2	<0.001	R
		Mixed NMs	2	0.04	−0.90,0.98	0.934	76.4	0.040	R
		MWCNTs	1	1.39	0.08,2.70	0.038	-	-	R
8-Isprostane	NM	SiO_2NPs	3	0.39	0.09,0.70	0.012	0.0	0.848	F
	type	Graphene	1	−0.69	−1.72,0.33	0.186	-	-	R
		CNT	1	0.69	0.20,1.18	0.006	-	-	R
		TiO_2NPs	7	2.37	1.04,3.70	<0.001	95.4	<0.001	R
		Mixed NMs	13	0.74	0.41,1.06	<0.001	72.7	<0.001	R
		ITONPs	1	1.74	1.20,2.29	<0.001	-	-	R
	Sample	EBC	16	1.17	0.64,1.69	<0.001	93.0	<0.001	R
	source	Urine	10	1.00	0.62,1.39	<0.001	58.6	0.010	R
8-isoPGF2a	NM	CNT	2	0.66	0.32,1.01	<0.001	0.0	0.328	F
	type	TiO_2NPs	2	0.70	0.37,1.03	<0.001	11.5	0.288	F
		SiO_2NPs	2	0.60	0.28,0.91	<0.001	0.0	0.698	F
		Mixed NMs	28	1.35	0.88,1.82	<0.001	95.3	<0.001	R
		IONPs	1	3.36	2.18,4.53	<0.001	-	-	R
	Sample	EBC	20	1.25	0.81,1.69	<0.001	94.3	<0.001	R

	source	Urine	9	0.35	−0.33,1.02	0.315	91.4	<0.001	R
		Blood	6	2.74	0.56,4.93	0.014	97.2	<0.001	R
8-OHdG	NM	SiO_2NPs	6	0.53	0.16,0.91	0.005	74.2	0.002	R
	type	Graphene	1	−0.29	−1.29,0.71	0.568	-	-	R
		Mixed NMs	55	0.92	0.69,1.16	<0.001	92.4	<0.001	R
		ITONPs	5	1.92	1.22,2.62	<0.001	91.8	<0.001	R
		IONPs	2	3.46	−3.39,10.31	0.322	97.6	<0.001	R
		TiO_2NPs	6	1.00	0.42,1.59	0.001	88.3	<0.001	R
	Sample	EBC	10	4.13	2.73,5.53	<0.001	89.5	<0.001	R
	source	Urine	35	0.67	0.42,0.93	<0.001	93.0	<0.001	R
		Blood	30	0.65	0.36,0.95	<0.001	94.5	<0.001	R
8-OHG	NM	IONPs	1	7.23	5.13,9.34	<0.001	-	-	R
	type	Mixed NMs	27	2.30	1.58,3.03	<0.001	94.4	<0.001	R
		TiO_2NPs	5	5.71	4.31,7.12	<0.001	81.0	<0.001	R
	Sample	EBC	14	4.60	2.92,6.27	<0.001	97.0	<0.001	R
	source	Urine	13	1.19	0.49,1.89	0.001	88.1	<0.001	R
		Blood	6	3.49	1.84,5.14	<0.001	94.7	<0.001	R
3-ClTyr	NM	IONPs	1	4.17	2.82,5.25	<0.001	-	-	R
	type	TiO_2NPs	5	5.79	3.63,7.95	<0.001	92.0	<0.001	R
		Mixed NMs	2	1.06	0.58,1.54	<0.001	0.0	0.969	F
5-OHMeU	NM	IONPs	1	4.52	3.08,5.95	<0.001	-	-	R
	type	Mixed NMs	27	1.55	0.85,2.25	<0.001	94.6	<0.001	R
		TiO_2NPs	5	3.23	1.79,4.67	<0.001	89.5	<0.001	R
	Sample	EBC	14	3.10	1.87,4.33	<0.001	95.5	<0.001	R
	source	Urine	13	0.37	−0.33,1.08	0.299	89.3	<0.001	R
		Blood	6	2.66	0.92,4.40	0.003	96.0	<0.001	R
o-Tyr	NM	IONPs	1	4.53	3.10,5.97	<0.001	-	-	R
	type	Mixed NMs	27	1.47	0.85,2.09	<0.001	93.4	<0.001	R
		TiO_2NPs	5	3.10	2.14,4.06	<0.001	77.6	0.001	R
	Sample	EBC	14	2.80	1.91,3.69	<0.001	92.5	<0.001	R
	source	Urine	13	0.44	−0.11,0.99	0.119	83.3	<0.001	R
		Blood	6	2.76	0.68,4.84	0.009	96.9	<0.001	R
3-NOTyr	NM	IONPs	1	4.81	3.31,6.31	<0.001	-	-	R
	type	Mixed NMs	5	4.01	2.77,5.26	<0.001	83.3	<0.001	R
		TiO_2NPs	20	2.17	1.21,3.14	<0.001	96.2	<0.001	R
	Sample	EBC	14	3.40	2.07,4.74	<0.001	96.1	<0.001	R
	source	Blood	6	2.21	0.15,4.28	0.036	96.9	<0.001	R
		Urine	6	1.36	−0.19,2.90	0.086	95.7	<0.001	R
Aldehydes	NM	IONPs	7	4.08	2.46,5.70	<0.001	91.3	<0.001	R
C6-12	type	TiO_2NPs	35	3.63	2.91,4.36	<0.001	94.0	<0.001	R
		Mixed NMs	18	2.86	1.54,4.17	<0.001	97.2	<0.001	R
	Sample	EBC	48	3.66	3.02,4.30	<0.001	94.4	<0.001	R
	source	Blood	6	4.89	1.08,8.71	0.012	98.1	<0.001	R
		Urine	6	0.84	−0.67,2.35	0.274	95.8	<0.001	R

NMs, nanomaterials; NPs, nanoparticles; SiO_2NPs, silica oxide nanoparticles; IONPs, iron oxide nanoparticles; TiO_2NPs, titanium dioxide nanoparticles; ITONPs, indium tin oxide nanoparticles; MWCNTs, multi-walled carbon nanotubes; EBC, exhaled breath condensate; MDA, malonaldehyde; SOD, superoxide dismutase; GPx, glutathione peroxidase; HNE, 4-hydroxy-2-nonhenal; HHE, 4-hydroxy-trans-hexenale; 8-OHdG, 8-hydroxydeoxyguanosine; 8-OHG, 8-hydroxyguanine; 8-isoPGF2a, 8-isoProstaglandin F2α; 5-OHMeU, hydroxymethyl uracil; o-Tyr, o-tyrosine; 3-ClTyr, 3-chlorotyrosine; 3-NOTyr, nitrotyrosine; SMD, standardized mean difference; CI, confidence interval; F, fixed-effects; R, random-effects; p_H-Value, significance for heterogeneity; p_A-Value, significance for associations.
