# Supplementary material for: Oxidative Stress and Inflammatory Biomarkers for Populations with Occupational Exposure to Nanomaterials: A Systematic Review and Meta-Analysis
Source: Antioxidants (Basel). 2022 Nov 4;11(11):2182. doi: 10.3390/antiox11112182 (PMC9687069; doi:10.3390/antiox11112182)
Supplement: Supplementary file 1 [file antioxidants-11-02182-s001.zip › Table S3.pdf]

Table S3. Subgroup analysis for inflammatory biomarkers

Variables			No.	SMD	95%CI	p_A-Value	I^2	p_H-Value	Model
FENO	NM type	SiO_2NPs	1	0.45	−0.55,1.46	0.379	-	-	R
		Graphene	1	0.40	−0.60,1.41	0.432	-	-	R
		Mixed NMs	12	0.07	−0.09,0.24	0.373	53.0	0.016	R
		TiO_2NPs	3	2.58	1.26,3.90	<0.001	85.7	0.001	R
IL-1β	NM type	Carbon black NPs	2	1.12	0.90,1.34	<0.001	0.0	0.941	F
		TiO_2NPs	1	0.05	−0.26,0.35	0.766	-	-	F
		MWCNTs	2	4.97	1.58,8.37	0.004	84.9	0.010	R
	Sample source	Blood	4	1.17	0.40,1.93	0.003	93.5	<0.001	R
		Sputum	1	6.84	4.56,9.12	<0.001	-	-	R
IL-4	NM type	Mixed NMs	2	0.07	−0.37,0.50	0.765	0.0	0.944	F
		MWCNTs	2	4.66	2.64,6.68	<0.001	63.7	0.097	R
	Sample source	EBC	2	0.07	−0.37,0.50	0.765	0.0	0.944	F
		Sputum	1	5.81	3.83,7.80	<0.001	-	-	F
		Blood	1	3.74	2.31,5.17	<0.001	-	-	F
IL-5	NM type	MWCNTs	2	3.08	0.94,5.22	0.005	80.7	0.023	R
		Mixed NMs	2	0.03	−0.41,0.46	0.903	0.0	0.900	F
	Sample source	Sputum	1	4.25	2.69,5.82	<0.001	-	-	F
		Blood	1	2.06	1.01,3.12	<0.001	-	-	F
		EBC	2	0.03	−0.41,0.46	0.903	0.0	0.900	F
IL-6	NM type	SiO_2NPs	1	−0.10	−1.10,0.89	0.842	-	-	R
		Graphene	1	−1.84	−3.04,−0.65	0.002	-	-	R
		Mixed NMs	14	0.07	−0.08,0.21	0.369	57.9	0.004	R
		Carbon black NPs	2	1.73	1.36,2.11	<0.001	59.5	0.116	R
		TiO_2NPs	1	0.88	0.56,1.19	<0.001	-	-	R
		MWCNTs	1	6.36	4.22,8.51	<0.001	-	-	R
	Sample source	Blood	19	0.22	−0.07,0.52	0.142	91.7	<0.001	R
		Sputum	1	6.36	4.22,8.51	<0.001	-	-	R
IL-8	NM type	SiO_2NPs	1	0.05	−0.95,1.04	0.930	-	-	F
		Graphene	1	−0.30	−1.30,0.70	0.555	-	-	F
		Mixed NMs	4	−1.44	−1.94,−0.93	<0.001	18.5	0.298	F
		Carbon black NPs	2	0.58	0.38,0.79	<0.001	0.0	0.948	F
		TiO_2NPs	1	0.87	0.55,1.19	<0.001	-	-	F
		MWCNTs	2	3.00	0.87,5.13	0.006	81.0	0.022	R
	Sample source	Blood	10	−0.15	−0.71,0.40	0.588	89.7	<0.001	R
		Sputum	1	4.17	2.63,5.71	<0.001	-	-	R
IL-10	NM type	TiO_2NPs	1	0.00	−0.30,0.30	1.000	-	-	F
		Carbon black NPs	2	−0.07	−0.50,0.37	0.758	0.0	0.792	F
		MWCNTs	1	3.99	2.50,5.48	<0.001	-	-	F

	Sample source	Blood	2	1.93	−1.98,5.83	0.334	96.2	<0.001	R
		EBC	2	−0.07	−0.50,0.37	0.758	0.0	0.792	F
TNF-α	NM type	Mixed NMs	10	1.06	0.56,1.56	<0.001	72.1	<0.001	R
		Carbon black NPs	2	1.99	1.74,2.24	<0.001	0.0	0.519	F
		TiO_2NPs	1	0.72	0.40,1.03	<0.001	-	-	F
		MWCNTs	2	5.04	3.78,6.30	<0.001	0.0	0.517	F
	Sample source	Blood	8	1.50	0.81,2.18	<0.001	91.5	<0.001	R
		Nasal lavage	4	1.25	0.34,2.16	0.007	75.2	0.007	R
		EBC	2	1.08	0.61,1.55	<0.001	0.0	0.712	F
		Sputum	1	5.51	3.61,7.42	<0.001	-	-	F
CC16	NM type	MWCNTs	6	−0.45	−0.86,−0.04	0.032	60.2	0.028	R
		Mixed NMs	14	−0.01	−0.10,0.08	0.816	12.6	0.316	F
		TiO_2NPs	1	−0.22	−0.53,0.08	0.151	-	-	F
SP-A	NM type	MWCNTs	6	0.07	−0.33,0.46	0.740	0.0	0.734	F
		TiO_2NPs	1	−0.13	−0.43,0.18	0.415	-	-	F
SP-D	NM type	MWCNTs	6	0.20	−0.20,0.60	0.332	47.2	0.092	F
		TiO_2NPs	1	−0.23	−0.54,0.07	0.130	-	-	F
CRP	NM type	Mixed NMs	16	0.05	−0.04,0.14	0.284	36.0	0.075	F
		TiO_2NPs	1	0.24	−0.07,0.54	0.127	-	-	F
		Carbon black NPs	1	1.71	1.37,2.04	<0.001	-	-	F
NF-κB	Sample source	Blood	14	−0.09	−0.22,0.05	0.230	54.2	0.008	R
		EBC	14	−0.01	−0.16,0.14	0.936	61.7	0.001	R
VCAM	NM type	Mixed NMs	14	0.06	−0.03,0.15	0.216	48.3	0.022	F
		TiO_2NPs	1	0.23	−0.08,0.53	0.144	-	-	F
ICAM	NM type	Mixed NMs	14	0.15	0.01,0.28	0.031	52.3	0.012	R
		TiO_2NPs	1	0.47	0.16,0.77	0.003	-	-	R
		MWCNTs	6	1.52	0.94,2.10	<0.001	38.5	0.150	F
LT-B4	NM type	Mixed NMs	2	0.44	0.01,0.88	0.048	0.0	0.834	F
		TiO_2NPs	5	3.32	1.21,5.43	0.002	97.3	<0.001	R
	Sample source	EBC	5	3.81	1.38,6.24	0.002	97.3	<0.001	R
		Urine	2	−0.17	−0.52,0.17	0.323	0.0	0.368	F
LT-C4	NM type	Mixed NMs	2	0.08	−0.35,0.52	0.703	0.0	0.838	F
		TiO_2NPs	5	1.76	−0.13,3.64	0.068	97.2	<0.001	R
	Sample source	EBC	5	2.11	0.26,3.97	0.026	96.4	<0.001	R
		Urine	2	−0.76	−1.78,0.26	0.143	87.5	0.005	R
LT-D4	NM type	Mixed NMs	2	0.20	−0.24,0.63	0.370	0.0	0.486	F
		TiO_2NPs	5	1.43	−0.36,3.22	0.117	97.0	<0.001	R
	Sample source	EBC	5	1.82	0.48,3.16	0.008	93.6	<0.001	R
		Urine	2	−0.81	−1.91,0.28	0.146	89.1	0.002	R
LT-E4	NM type	Mixed NMs	2	0.50	0.06,0.94	0.025	0.0	0.938	F
		TiO_2NPs	5	2.17	−0.06,4.40	0.056	97.0	<0.001	R
	Sample	EBC	5	2.67	0.96,4.38	0.002	95.1	<0.001	R

source	Urine	2	-0.79	−1.84,0.26	0.142	88.2	0.004	R

NMs, nanomaterials; NPs, nanoparticles; SiO2NPs, silica oxide nanoparticles; IONPs, iron oxide nanoparticles; TiO2NPs, titanium dioxide nanoparticles; ITONPs, indium tin oxide nanoparticles; MWCNTs, multi-walled carbon nanotubes; EBC: exhaled breath condensate; FENO, fraction of exhaled nitric oxide; IL, interleukin; TNF, tumor necrosis factor; NF-κB, nuclear factor kappa-B; CRP, C-reactive protein; CC16, club cell secretory protein 16; SP-A, surfactant protein A; SP-D, surfactant protein D; VCAM, vascular cell adhesion molecule; ICAM, intercellular adhesion molecule; LT-B4, leukotriene B4; LT-C4, leukotriene C4; LT-D4, leukotriene D4; LT-E4, leukotriene E4; SMD, standardized mean difference; CI, confidence interval; F, fixed-effects; R, random-effects; p_H-Value, significance for heterogeneity; p_A-Value, significance for associations.
